# Supplementary material for: Conjunctive Analyses of BSA-Seq and BSR-Seq to Identify Candidate Genes Controlling the Black Lemma and Pericarp Trait in Barley
Source: Int J Mol Sci. 2023 May 30;24(11):9473. doi: 10.3390/ijms24119473 (PMC10253945; doi:10.3390/ijms24119473)
Supplement: Supplementary file 1 [file ijms-24-09473-s001.zip › Supplementary Method S1.pdf]

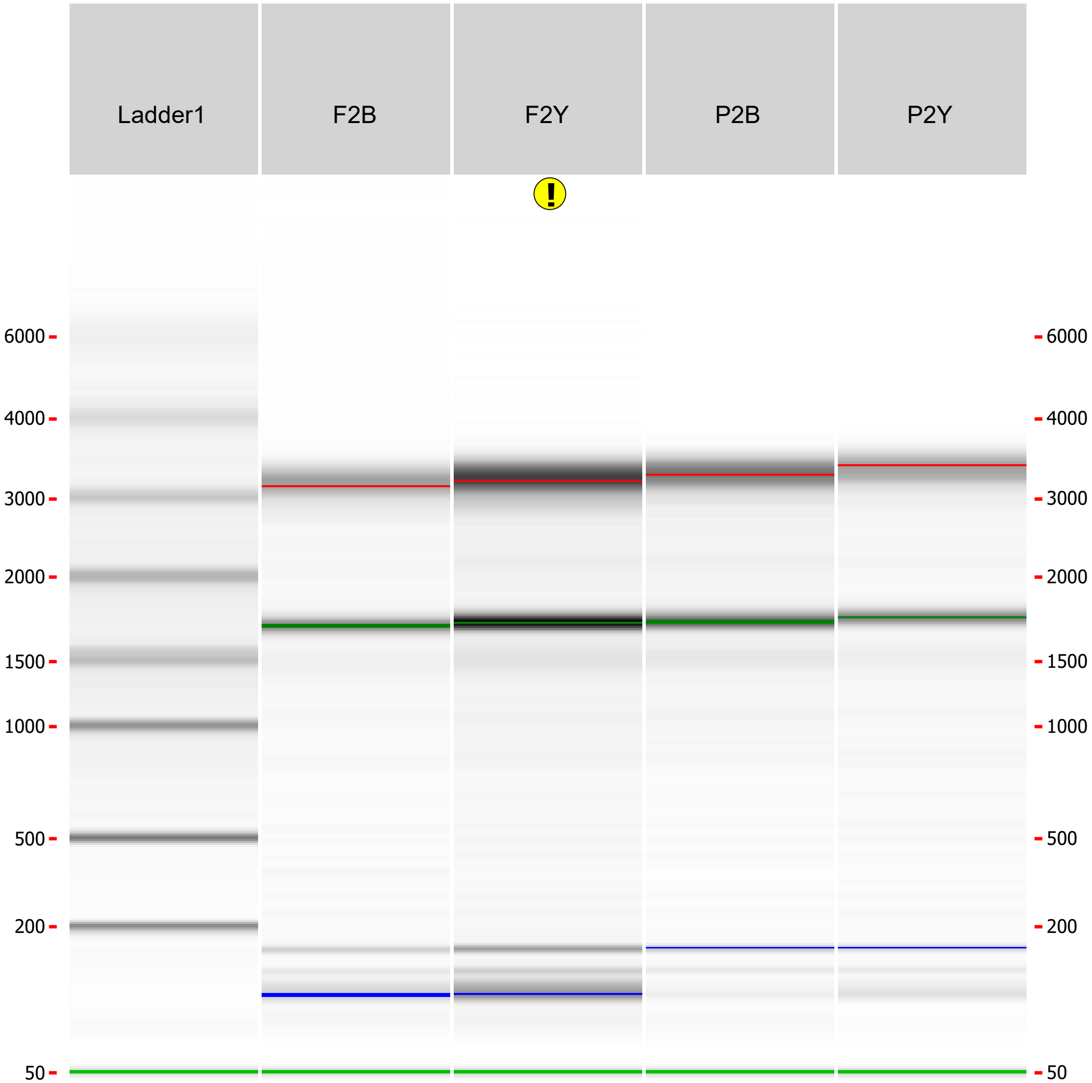

LabChip GX Software Version 5.4.2222.0
Copyright © 2017 PerkinElmer,Inc.
Datafile Created 2020/6/20 1:33:40 with S/W Version: 1.7.819.0
Analysis Version: 1
Chip Y521B-0564N-03 (Expiry: 2022/1/6)
20220620-RNA2.gxd

Modified: 2022/6/20 14:28:48 by DESKTOP-76N0R9H\dell
Printed: 2023/5/8 11:14:09
Firmware Version: 1.02.0.37277L_45
Instrument: WINDOWS-H0697IN (S/N GT1810N0855)
Operator: qhzhang

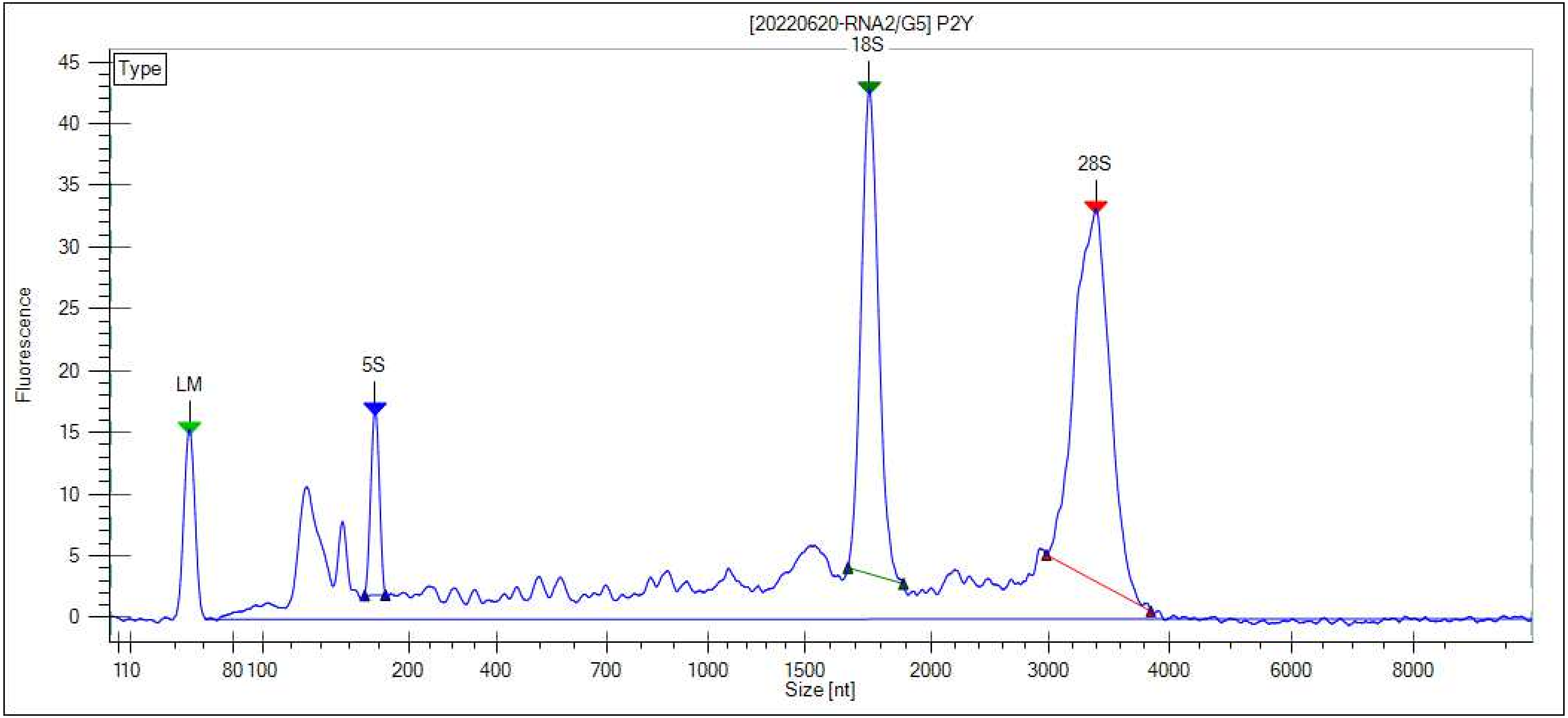

Copyright © 2017 PerkinElmer,Inc.

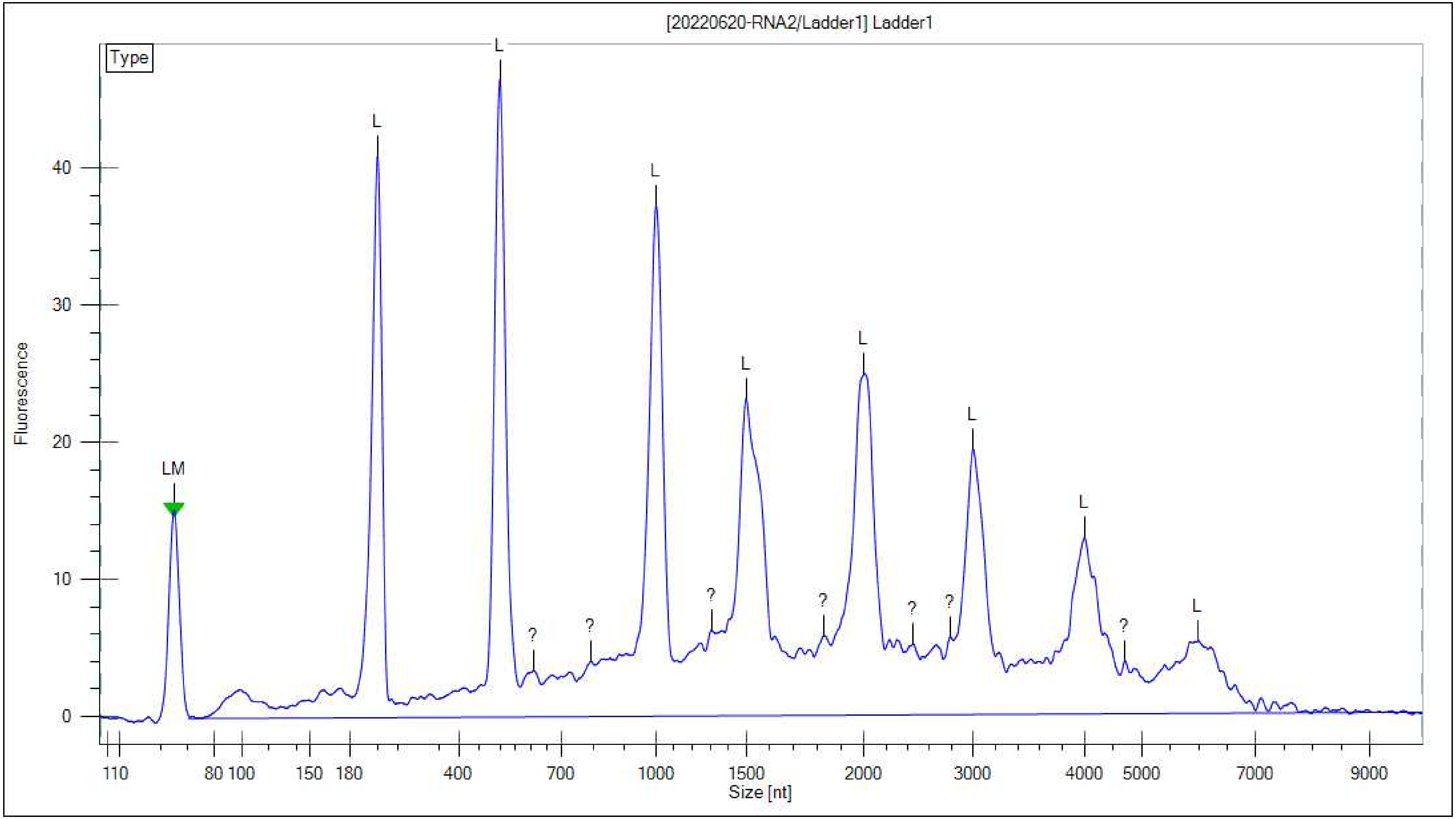

LabChip GX Software Version 5.4.2222.0
Copyright © 2017 PerkinElmer,Inc.
Datafile Created 2020/6/20 1:33:40 with S/W Version: 1.7.819.0
Analysis Version: 1
Chip Y521B-0564N-03 (Expiry: 2022/1/6)
20220620-RNA2.gxd

Modified: 2022/6/20 14:28:48 by DESKTOP-76N0R9H\dell
Printed: 2023/5/8 11:14:09
Firmware Version: 1.02.0.37277L_45
Instrument: WINDOWS-H0697IN (S/N GT1810N0855)
Operator: qhzhang

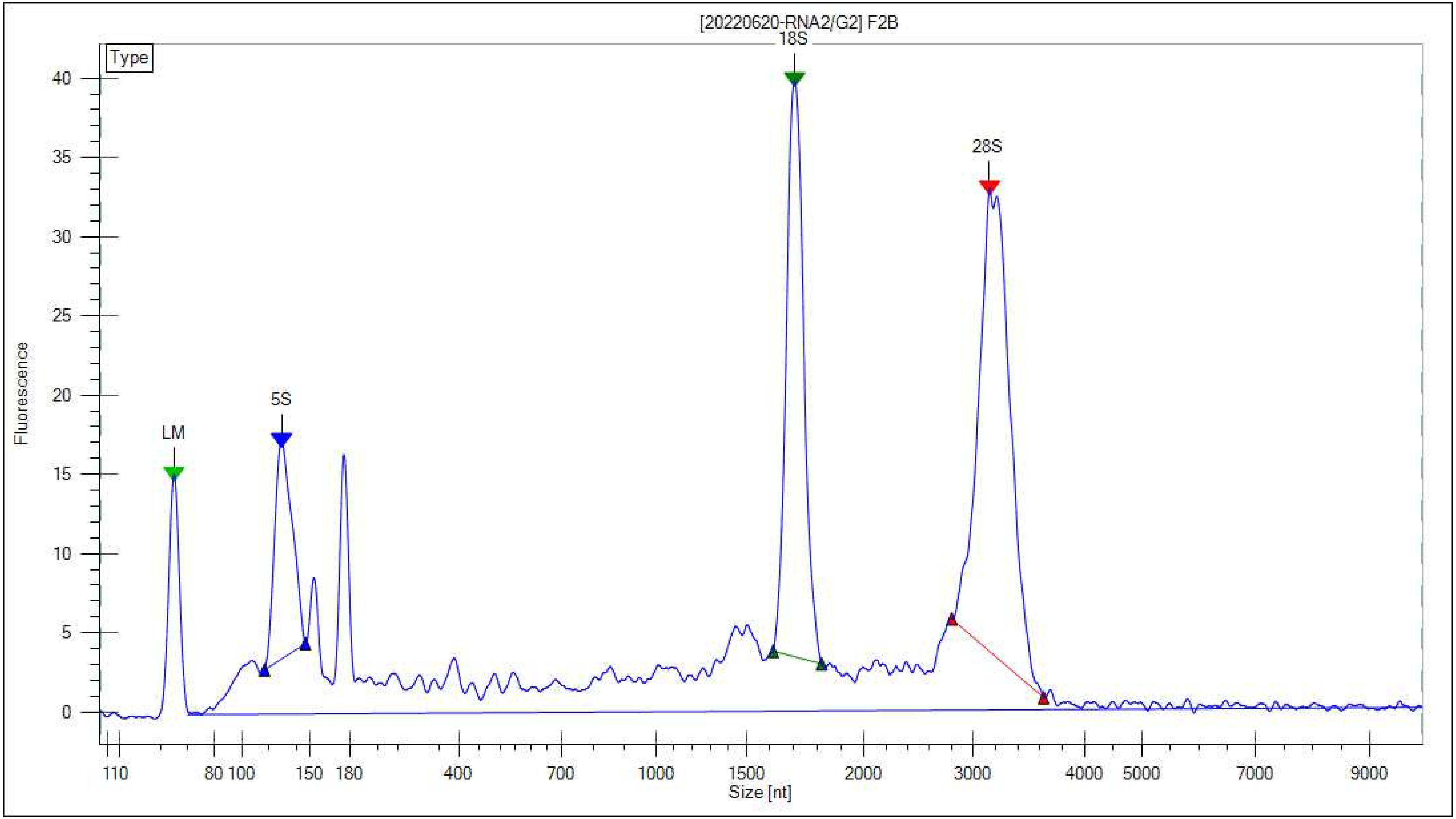

LabChip GX Software Version 5.4.2222.0
Copyright © 2017 PerkinElmer,Inc.
Datafile Created 2020/6/20 1:33:40 with S/W Version: 1.7.819.0
Analysis Version: 1
Chip Y521B-0564N-03 (Expiry: 2022/1/6)
20220620-RNA2.gxd

Modified: 2022/6/20 14:28:48 by DESKTOP-76N0R9H\dell
Printed: 2023/5/8 11:14:09
Firmware Version: 1.02.0.37277L_45
Instrument: WINDOWS-H0697IN (S/N GT1810N0855)
Operator: qhzhang

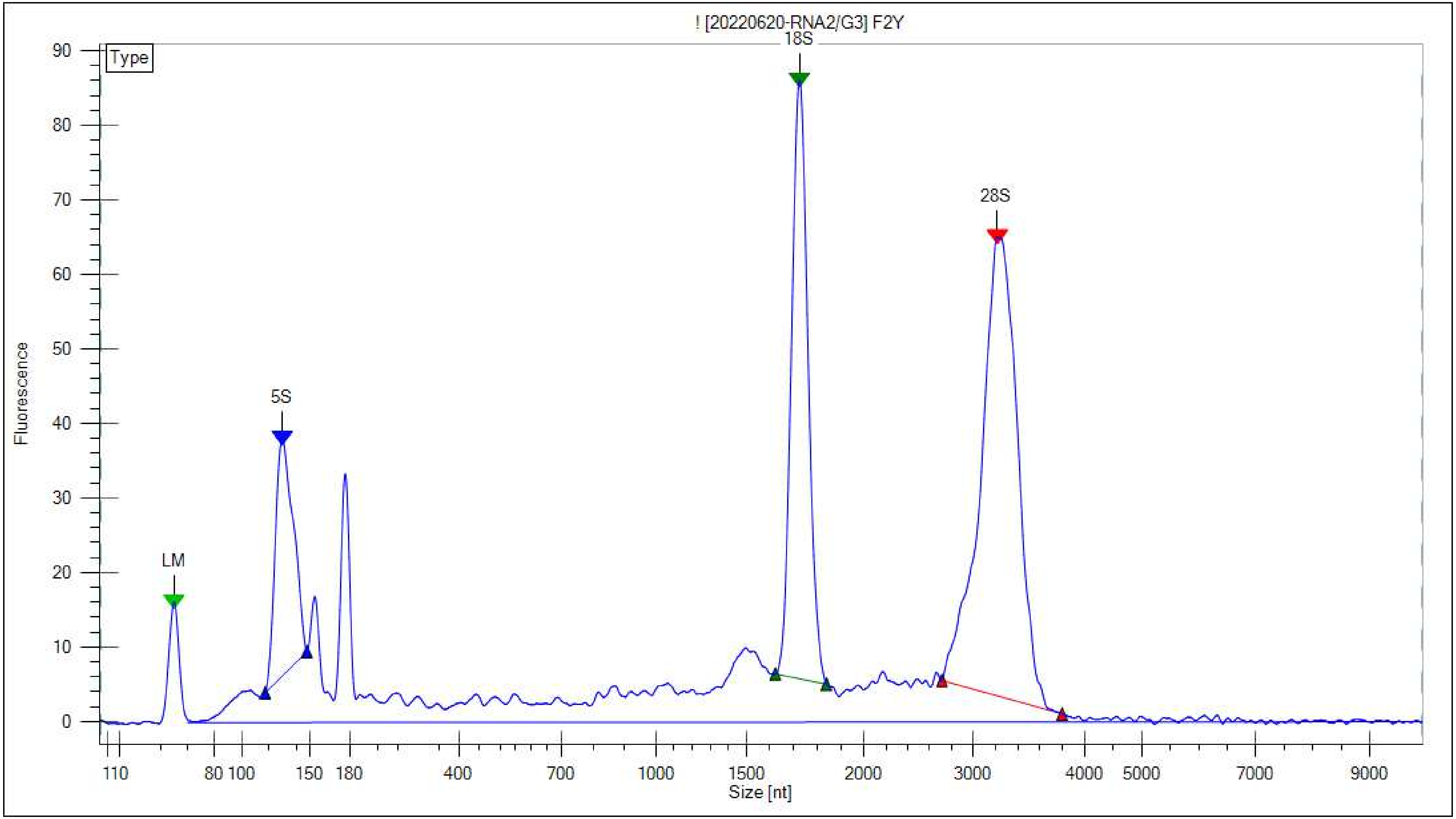

LabChip GX Software Version 5.4.2222.0
Copyright © 2017 PerkinElmer,Inc.
Datafile Created 2020/6/20 1:33:40 with S/W Version: 1.7.819.0
Analysis Version: 1
Chip Y521B-0564N-03 (Expiry: 2022/1/6)
20220620-RNA2.gxd

Modified: 2022/6/20 14:28:48 by DESKTOP-76N0R9H\dell
Printed: 2023/5/8 11:14:09
Firmware Version: 1.02.0.37277L_45
Instrument: WINDOWS-H0697IN (S/N GT1810N0855)
Operator: qhzhang

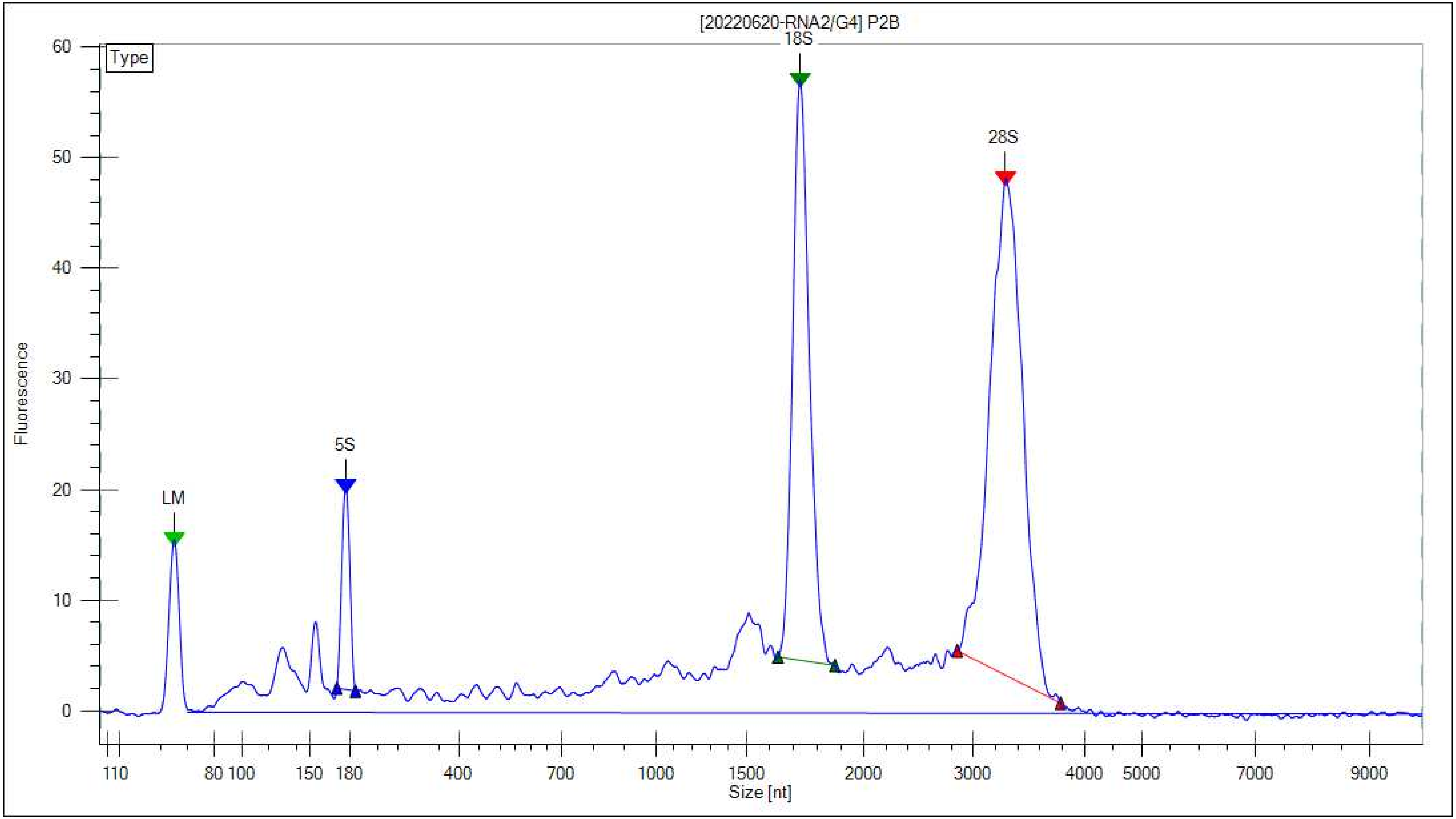

LabChip GX Software Version 5.4.2222.0
Copyright © 2017 PerkinElmer,Inc.
Datafile Created 2020/6/20 1:33:40 with S/W Version: 1.7.819.0
Analysis Version: 1
Chip Y521B-0564N-03 (Expiry: 2022/1/6)
20220620-RNA2.gxd

Modified: 2022/6/20 14:28:48 by DESKTOP-76N0R9H\dell
Printed: 2023/5/8 11:14:10
Firmware Version: 1.02.0.37277L_45
Instrument: WINDOWS-H0697IN (S/N GT1810N0855)
Operator: qhzhang

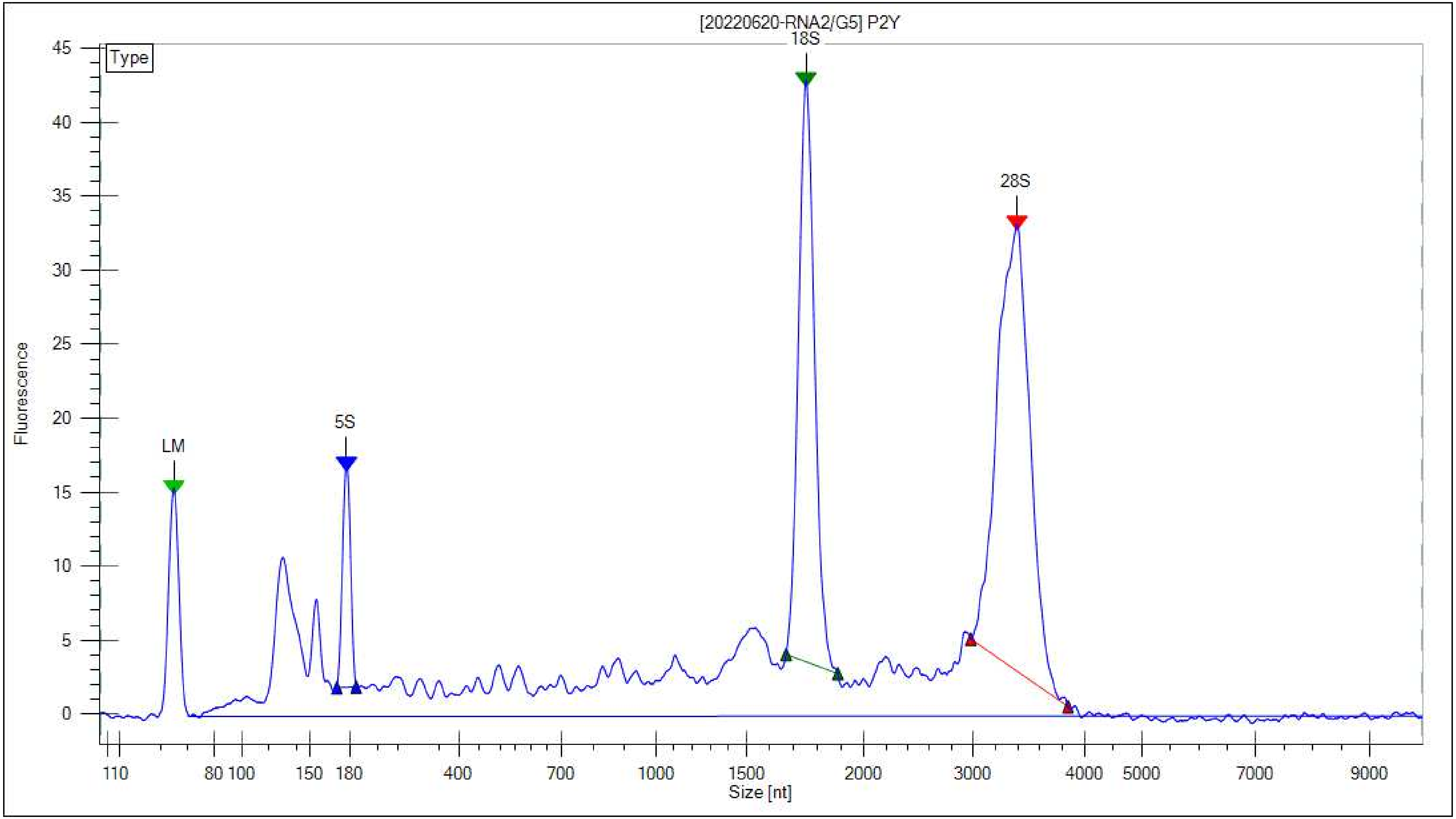

Copyright © 2017 PerkinElmer,Inc.

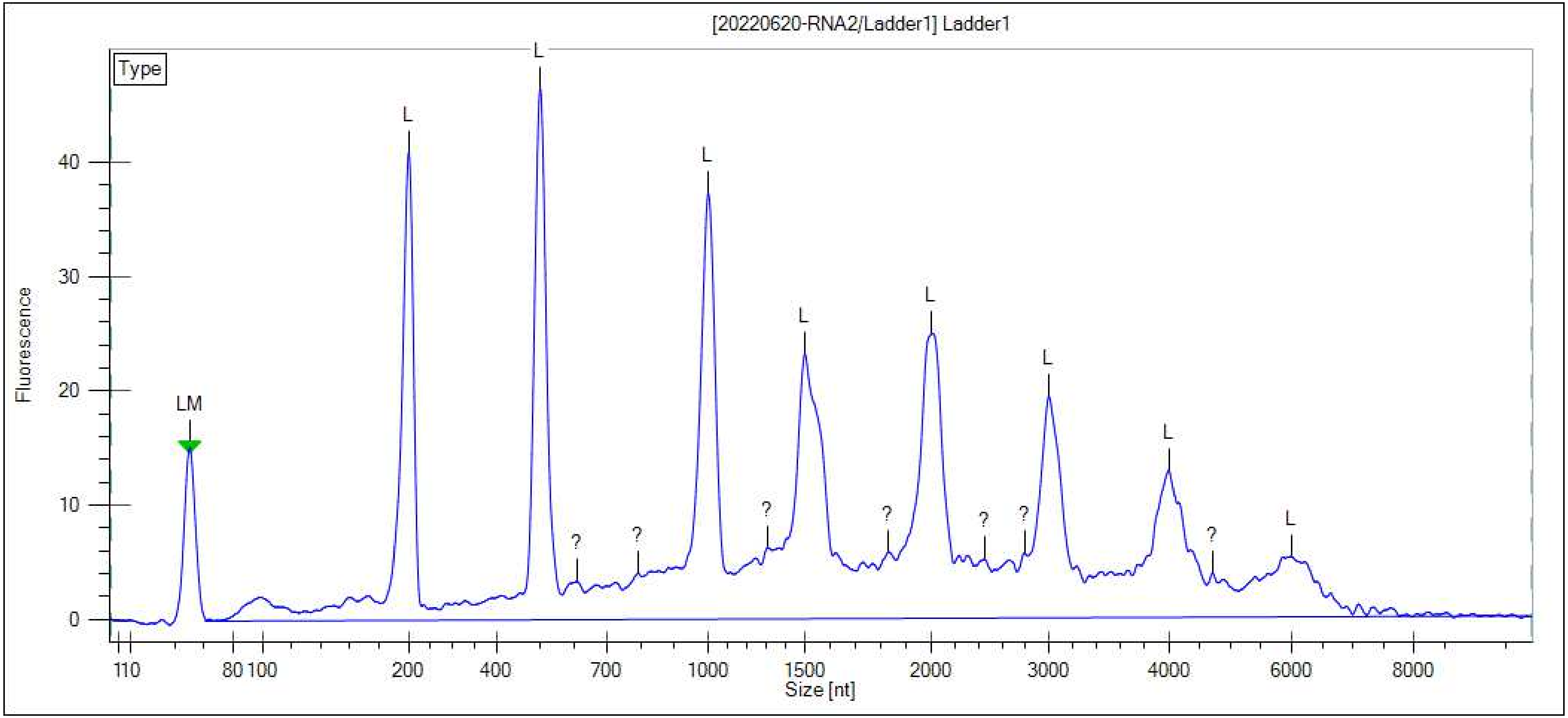
[20220620-RNA2/Ladder1] Ladder1
Type
Fluorescence
LM
L
?
0
10
20
30
40
110
80 100
200
400
700
1000
1500
2000
3000
4000
6000
8000
Size [nt]

Plate Name	Well Label	Sample Name	Peak Count	Total Conc. (ng/ul)	RNA Area	rRNA Area Ratio [28S/18S]	rRNA Height Ratio [28S/18S]	rRNA Fast Area Ratio	RNA Quality Score	5S Area	5S % Total	18S Area	18S % Total	28S Area	28S % Total
20220620-RNA2	Ladder01	Ladder1		83.30	290.09										

Well Label	Sample Name	Type	Size [nt]	Migration Time (sec) - Start	Migration Time (sec) - End	RNA Fragment	Fragment Area	% of Total Area	Fragment Start(sec)	Fragment End(sec)
Ladder01	Ladder1	LM	50							
Ladder01	Ladder1	L	200							
Ladder01	Ladder1	L	500							
Ladder01	Ladder1	?	609							
Ladder01	Ladder1	?	791							
Ladder01	Ladder1	L	1000							
Ladder01	Ladder1	?	1307							
Ladder01	Ladder1	L	1500							
Ladder01	Ladder1	?	1830							
Ladder01	Ladder1	L	2000							
Ladder01	Ladder1	?	2452							
Ladder01	Ladder1	?	2793							
Ladder01	Ladder1	L	3000							
Ladder01	Ladder1	L	4000							
Ladder01	Ladder1	?	4712							
Ladder01	Ladder1	L	6000							

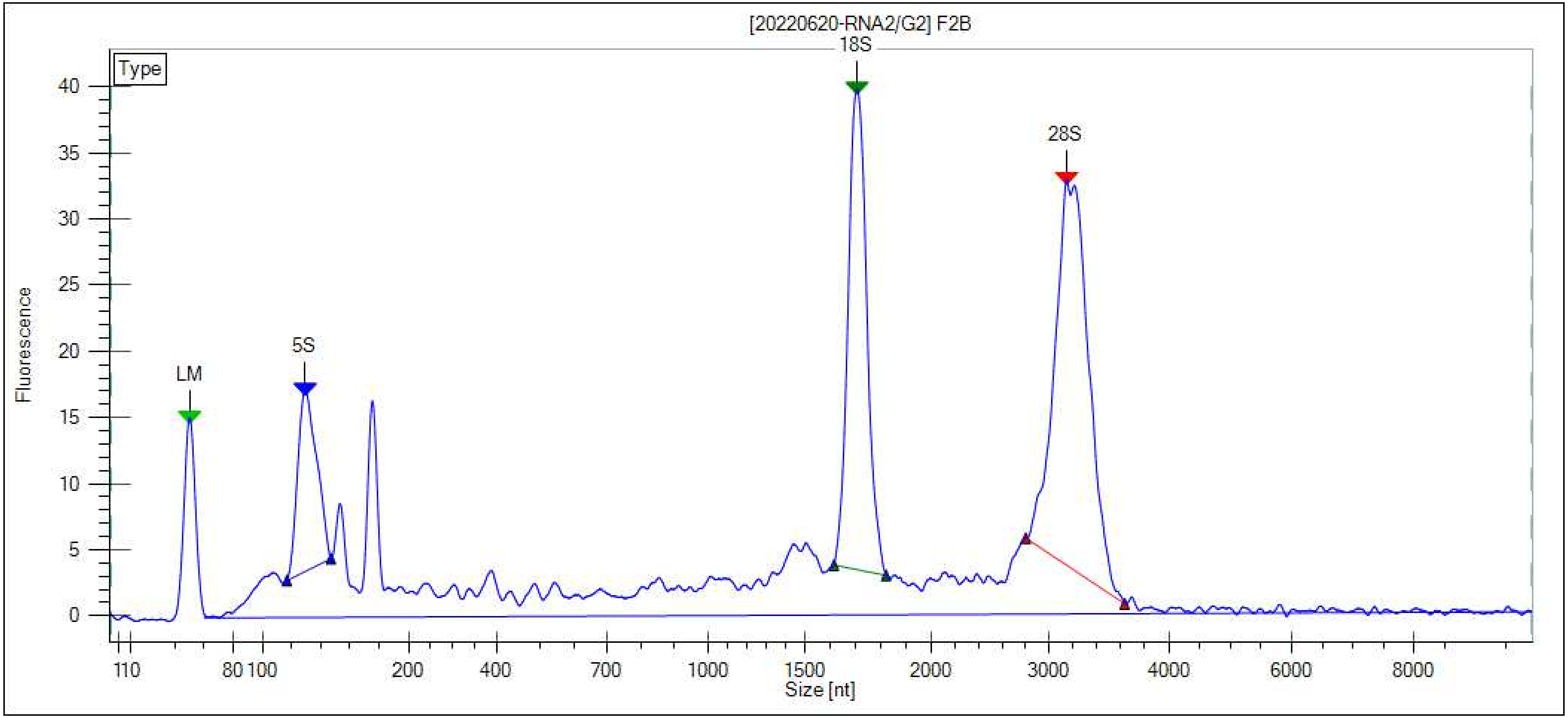
[20220620-RNA2/G2] F2B
Type
18S
28S
5S
LM
Fluorescence
0
5
10
15
20
25
30
35
40
110
80 100
200
400
700
1000
1500
2000
3000
4000
6000
8000
Size [nt]

Plate Name	Well Label	Sample Name	Peak Count	Total Conc. (ng/ul)	RNA Area	rRNA Area Ratio [28S/18S]	rRNA Height Ratio [28S/18S]	rRNA Fast Area Ratio	RNA Quality Score	5S Area	5S % Total	18S Area	18S % Total	28S Area	28S % Total
20220620-RNA2	G02	F2B	16	58.14	204.89	1.47	0.86	0.28	7.1	12.73	6.2%	33.18	16.2%	48.79	23.8%

Well Label	Sample Name	Type	Size [nt]	Migration Time (sec) - Start	Migration Time (sec) - End	RNA Fragment	Fragment Area	% of Total Area	Fragment Start(sec)	Fragment End(sec)
G02	F2B	LM	50							
G02	F2B		107							
G02	F2B	5S	129			5S	12.73	6.2%	29.43	31.28
G02	F2B		153							
G02	F2B		175							
G02	F2B		304							
G02	F2B		387							
G02	F2B		487							
G02	F2B		542							
G02	F2B		854							
G02	F2B		1010							
G02	F2B		1443							
G02	F2B	18S	1706			18S	33.18	16.2%	52.38	54.57
G02	F2B		2103							
G02	F2B		2801							
G02	F2B	28S	3151			28S	48.79	23.8%	60.43	64.58
G02	F2B		3694							

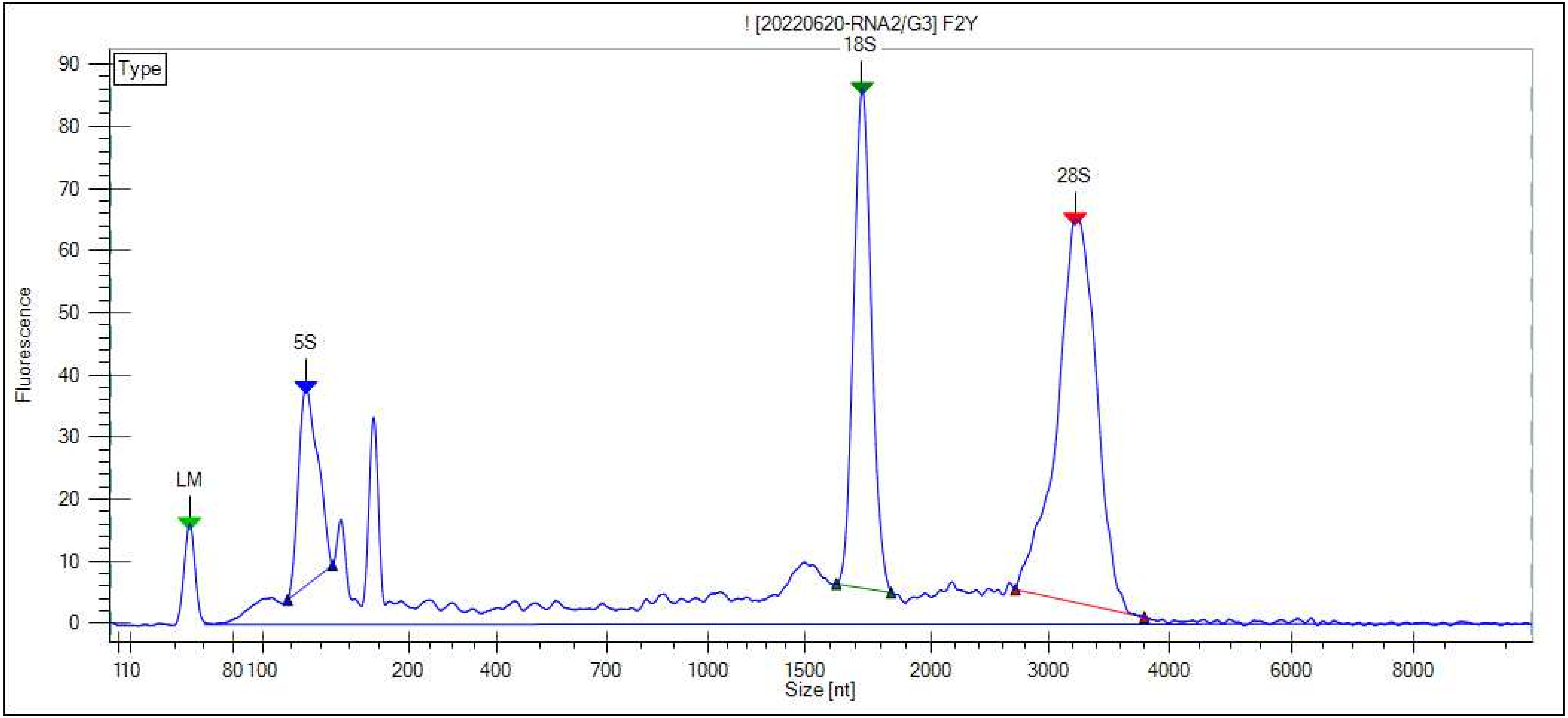
! [20220620-RNA2/G3] F2Y
Type
18S
28S
5S
LM
Fluorescence
90
80
70
60
50
40
30
20
10
0
110
80 100
200
400
700
1000
1500
2000
3000
4000
6000
8000
Size [nt]

Plate Name	Well Label	Sample Name	Peak Count	Total Conc. (ng/ul)	RNA Area	rRNA Area Ratio [28S/18S]	rRNA Height Ratio [28S/18S]	rRNA Fast Area Ratio	RNA Quality Score	5S Area	5S % Total	18S Area	18S % Total	28S Area	28S % Total
20220620-RNA2	G03	F2Y	22	113.05	400.39	1.71	0.77	0.25	[7.5]	30.04	7.5%	69.63	17.4%	119.16	29.8%

Well Label	Sample Name	Type	Size [nt]	Migration Time (sec) - Start	Migration Time (sec) - End	RNA Fragment	Fragment Area	% of Total Area	Fragment Start(sec)	Fragment End(sec)
G03	F2Y	LM	50							
G03	F2Y		100							
G03	F2Y	5S	130			5S	30.04	7.5%	29.47	31.35
G03	F2Y		154							
G03	F2Y		176							
G03	F2Y		246							
G03	F2Y		298							
G03	F2Y		442							
G03	F2Y		489							
G03	F2Y		546							
G03	F2Y		685							
G03	F2Y		815							
G03	F2Y		868							
G03	F2Y		1065							
G03	F2Y		1499							
G03	F2Y	18S	1725			18S	69.63	17.4%	52.48	54.78
G03	F2Y		2168							
G03	F2Y		2485							
G03	F2Y		2564							
G03	F2Y		2664							
G03	F2Y	28S	3222			28S	119.16	29.8%	60.00	65.42
G03	F2Y		6098							
G03	F2Y		6327							

Copyright © 2017 PerkinElmer,Inc.

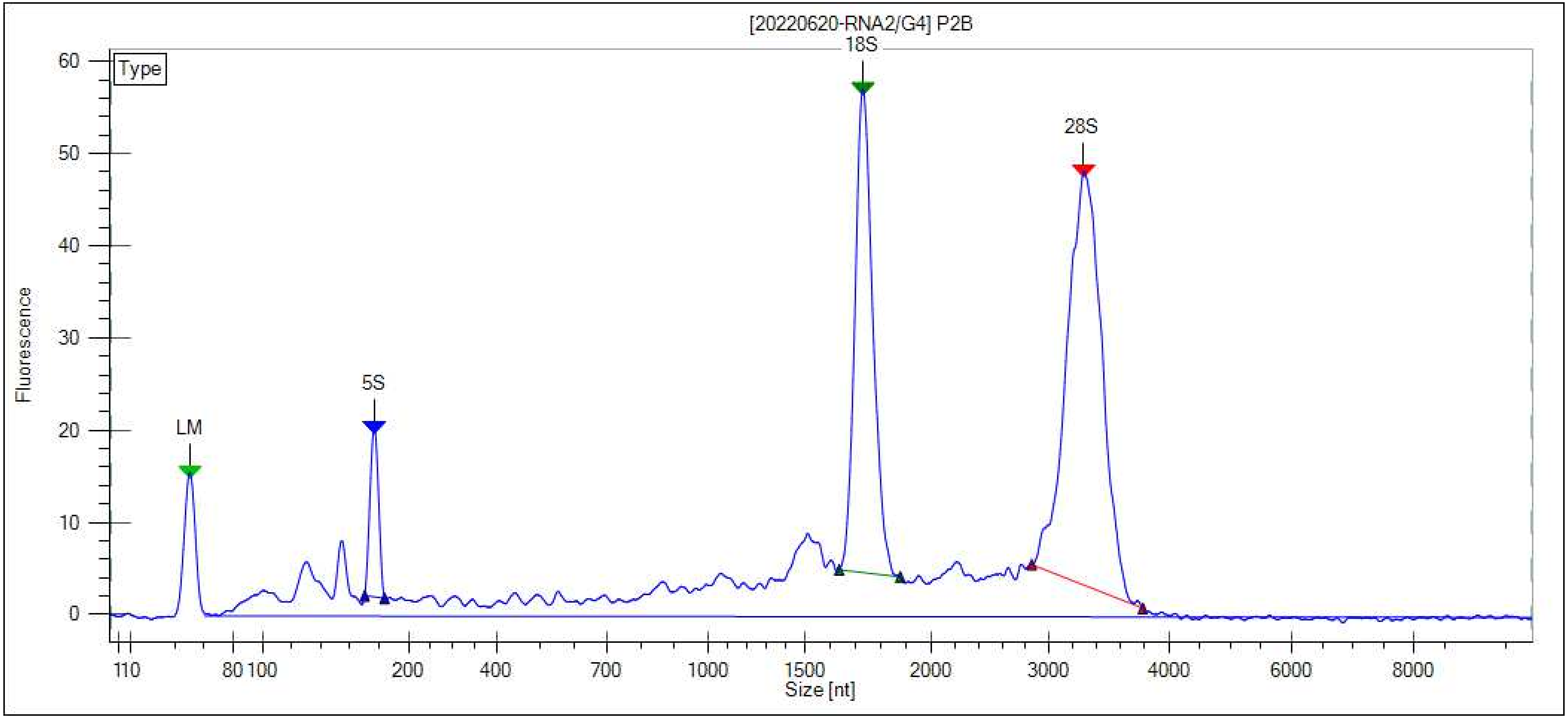

Copyright © 2017 PerkinElmer,Inc.

Plate Name	Well Label	Sample Name	Peak Count	Total Conc. (ng/ul)	RNA Area	rRNA Area Ratio [28S/18S]	rRNA Height Ratio [28S/18S]	rRNA Fast Area Ratio	RNA Quality Score	5S Area	5S % Total	18S Area	18S % Total	28S Area	28S % Total
20220620-RNA2	G04	P2B	17	73.82	254.58	1.69	0.86	0.21	7.8	7.68	3.0%	45.79	18.0%	77.26	30.3%

Well Label	Sample Name	Type	Size [nt]	Migration Time (sec) - Start	Migration Time (sec) - End	RNA Fragment	Fragment Area	% of Total Area	Fragment Start(sec)	Fragment End(sec)
G04	P2B	LM	50							
G04	P2B		100							
G04	P2B		130							
G04	P2B		154							
G04	P2B	5S	177			5S	7.68	3.0%	32.70	33.53
G04	P2B		307							
G04	P2B		444							
G04	P2B		494							
G04	P2B		552							
G04	P2B		863							
G04	P2B		1066							
G04	P2B		1510							
G04	P2B		1602							
G04	P2B	18S	1728			18S	45.79	18.0%	52.60	55.17
G04	P2B		2213							
G04	P2B		2651							
G04	P2B		2762							
G04	P2B	28S	3293			28S	77.26	30.3%	60.68	65.35

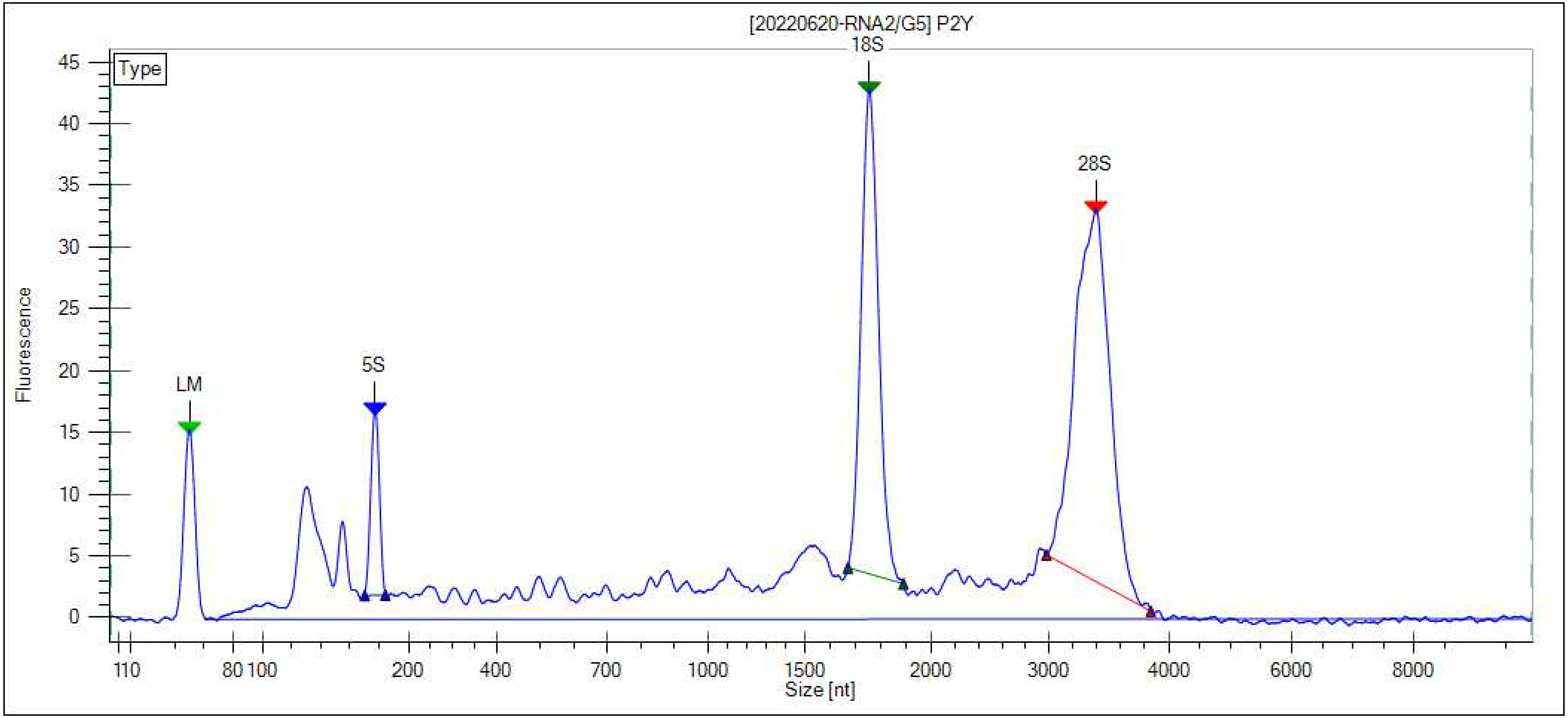

Copyright © 2017 PerkinElmer,Inc.

Plate Name	Well Label	Sample Name	Peak Count	Total Conc. (ng/ul)	RNA Area	rRNA Area Ratio [28S/18S]	rRNA Height Ratio [28S/18S]	rRNA Fast Area Ratio	RNA Quality Score	5S Area	5S % Total	18S Area	18S % Total	28S Area	28S % Total
20220620-RNA2	G05	P2Y	19	59.05	200.77	1.55	0.80	0.25	7.2	6.29	3.1%	33.08	16.5%	51.37	25.6%

Well Label	Sample Name	Type	Size [nt]	Migration Time (sec) - Start	Migration Time (sec) - End	RNA Fragment	Fragment Area	% of Total Area	Fragment Start(sec)	Fragment End(sec)
G05	P2Y	LM	50							
G05	P2Y		130							
G05	P2Y		155							
G05	P2Y	5S	177			5S	6.29	3.1%	32.70	33.57
G05	P2Y		247							
G05	P2Y		303							
G05	P2Y		351							
G05	P2Y		446							
G05	P2Y		498							
G05	P2Y		560							
G05	P2Y		694							
G05	P2Y		829							
G05	P2Y		880							
G05	P2Y		1106							
G05	P2Y		1522							
G05	P2Y	18S	1753			18S	33.08	16.5%	52.97	55.30
G05	P2Y		2192							
G05	P2Y		2317							
G05	P2Y		2929							
G05	P2Y	28S	3394			28S	51.37	25.6%	61.30	65.68
G05	P2Y	?								

Copyright © 2017 PerkinElmer,Inc.
